# A Green Route for High-Yield Production of Tetramethylpyrazine From Non-Food Raw Materials

**DOI:** 10.3389/fbioe.2021.792023

**Published:** 2022-01-25

**Authors:** Jing Li, Jian Lu, Zhilin Ma, Jianxiu Li, Xianrui Chen, Mengxue Diao, Nengzhong Xie

**Affiliations:** ^1^ Life Science and Technology College, State Key Laboratory for Conservation and Utilization of Subtropical Agro-bioresources, Guangxi University, Nanning, Guangxi, China; ^2^ State Key Laboratory of Non-food Biomass and Enzyme Technology, National Engineering Research Center for Non-food Biorefinery, Guangxi Biomass Engineering Technology Research Center, Guangxi Key Laboratory of Biorefinery, Guangxi Academy of Sciences, Nanning, Guangxi, China

**Keywords:** tetramethylpyrazine, non-food raw materials, synthetic biology, acetoin, metabolic engineering, green process

## Abstract

2,3,5,6-Tetramethylpyrazine (TMP) is an active pharmaceutical ingredient originally isolated from *Ligusticum wallichii* for curing cardiovascular and cerebrovascular diseases and is widely used as a popular flavoring additive in the food industry. Hence, there is a great interest in developing new strategies to produce this high-value compound in an ecological and economical way. Herein, a cost-competitive combinational approach was proposed to accomplish green and high-efficiency production of TMP. First, microbial cell factories were constructed to produce acetoin (3-hydroxy-2-butanone, AC), an endogenous precursor of TMP, by introducing a biosynthesis pathway coupled with an intracellular NAD^+^ regeneration system to the wild-type *Escherichia coli*. To further improve the production of (*R*)-AC, the metabolic pathways of by-products were impaired or blocked stepwise by gene manipulation, resulting in 40.84 g/L (*R*)-AC with a high optical purity of 99.42% in shake flasks. Thereafter, an optimal strain designated GXASR11 was used to convert the hydrolysates of inexpensive feedstocks into (*R*)-AC and achieved a titer of 86.04 g/L within 48 h in a 5-L fermenter under optimized fermentation conditions. To the best of our knowledge, this is the highest (*R*)-AC production with high optical purity (≥98%) produced from non-food raw materials using recombinant *E*. *coli*. The supernatant of fermentation broth was mixed with diammonium phosphate (DAP) to make a total volume of 20 ml and transferred to a high-pressure microreactor. Finally, 56.72 g/L TMP was obtained in 3 h via the condensation reaction with a high conversion rate (85.30%) under optimal reaction conditions. These results demonstrated a green and sustainable approach to efficiently produce high-valued TMP, which realized value addition of low-cost renewables.

## 1 Introduction

2,3,5,6-Tetramethylpyrazine (TMP), also known as ligustrazine, is a biologically active alkaloid, which occurs naturally in the rhizome of a Chinese traditional medicinal herb, Chuanxiong (*Ligusticum wallichii*) (Xiao et al., 2006; Chen et al., 2018). TMP is a colorless compound with pleasant roasted, nutty flavor characteristics, and is usually used as a flavor additive in the food and beverage industries (Xiao et al., 2014; Xu et al., 2018). More importantly, TMP is beneficial to human health and has been proven to have a variety of pharmacological activities. Many pharmacological studies have confirmed that it can prevent a series of diseases, including cardiovascular and cerebrovascular diseases, ischemic stroke, cancer and diabetes (Cao et al., 2015; Guo et al., 2016). Accordingly, studies on TMP have attracted considerable attention.

Currently, there exist three main approaches for TMP production, including extraction from medicinal plants, chemical synthesis, and biomanufacturing (Müller and Rappert, 2010; Jia et al., 2017a). Previous studies have concluded that TMP is one of the key ingredients of *Rhizoma Chuanxiong* (RC), however, it presents in trace amounts in RC ranging from lower than 0.10–11.75 μg/g (Huang et al., 2011; Chen et al., 2018; Yin et al., 2018). Therefore, the extraction of natural TMP is costly, inefficient and unsustainable (Zhu et al., 2010; Xiao et al., 2014; Jia et al., 2017a; Peng et al., 2020a). The chemical synthesis approach has become less desirable because of the stringent requirement of environmental protection, the current shortage of fossil fuels, and the consideration of manufacturing costs (Jia et al., 2017a). Compared with direct extraction and chemical synthesis, the bio-based route is more environmentally friendly and cost-efficient (Meng et al., 2015).

The compound acetoin (3-hydroxy-2-butanone, AC) is an indispensable flavor enhancer widely applied in foods, cosmetics and chemical synthesis (Li et al., 2018; Xiao et al., 2018) and also plays a pivotal role in TMP synthesis as a precursor (Demain et al., 1967; Xiao et al., 2014; Peng et al., 2020a; Zhong et al., 2020a). To improve the AC accumulation in microbial fermentation, various strategies have been employed, including screening of efficient natural producers of acetoin (Xiao et al., 2006; Zhu et al., 2010; Zhang et al., 2011; Yin et al., 2018; Zhong et al., 2020b), metabolic engineering (Vivijs et al., 2014; Xu et al., 2015; Jang et al., 2017; Li et al., 2018; Bae et al., 2021) and fermentation optimization (Xiao et al., 2007; Zhu and Xu 2010a; Zhang et al., 2012; Yuan et al., 2019).

Studies regarding TMP production from various carbon and nitrogen sources using biological approaches are summarized in Table 1. Most reports employed commercial glucose as the carbon source, while costly yeast extract, peptone and tryptone were used as the major nitrogen sources. Thus, it could be deduced that most biomanufacturing, which relied heavily on refined feedstocks, was still limited by relatively high costs, therefore not suitable for an economically feasible industrial fermentation process (Zhu et al., 2010; Xiao et al., 2014). To overcome the limitation, cheaper and readily available feedstocks and efficient fermentation systems are both required to be exploited for TMP production.

**TABLE 1:** Summary of studies on TMP production from different carbon and nitrogen sources.

Microorganisms	Carbon sources	Nitrogen sources	Source of ammonium ion	TMP (g/L)	References
*B*. *amyloliquefaciens* XJB-104	Wheat bran, distiller’s grains	Soybean meal	Amino acid	1.28	Zhang et al. (2019)
*B*. *coagulans* CICC 20138	Glucose	Yeast extract	DAP	2.54	Zhong et al. (2020a)
*B*. *licheniformis* BL1 (Engineered)	Glucose	Tryptone, yeast extract	ND^a^	44.77	Meng et al. (2016)
*B*. *licheniformis* BLC (Engineered)	Glucose, acetaldehyde	Tryptone, yeast extract	ND	47.26	Meng et al. (2020)
*B*. *subtilis* BJ3-2	Adlay	Adlay	ND	6.93^b^	Wen et al. (2020)
*B*. *subtilis* BS2	Glucose	Tryptone, yeast extract	ND	29.70	Meng et al. (2015)
*B*. *subtilis* CICC 10211	Glucose	Yeast extract, tryptone	DAP	8.34	Xiao et al. (2014)
*B*. *subtilis* CICC 20030	Rapeseed meal, glucose, wheat bran	Rapeseed meal, wheat bran	Amino acids	0.49	Hao et al. (2020)
*B*. *subtilis* CCTCC M 208,157	Glucose	Wheat bran	DAP	3.01^b^	Hao et al. (2013)
*B*. *subtilis* CCTCC M 208,157	Glucose	Yeast extract	DAP	7.43	Zhu and Xu (2010b)
*B*. *subtilis* CCTCC M 208,157	Glucose	Fish peptone, yeast extract	DAP	7.46	Zhu and Xu (2010a)
*B*. *subtilis* IFO 3013	Glucose, AC	Soybean peptone	Amino acid	2.50^b^	Besson et al. (1997)
*B*. *subtilis* IFO 3013	Soybean, acetoin	Soybean	L-Threonin	0.53	Larroche et al. (1999)
*B*. *subtilis* LB5	Glucose	Yeast extract, peptone	ND	10.69	Yin et al. (2018)
*B*. *subtilis* XZ1124	Glucose	Yeast extract, peptone	DAP	4.20	Zhu et al. (2010)
*Bacillus* sp. RX3-17	Glucose	Soytone	DAP	4.33	Xiao et al. (2006)
*Corynebacterium glutamicum* MB-1923	Glucose	Urea, N-Z-amine	DAP, ammonium sulphate	3.00	Demain et al. (1967)
*E*. *coli* BL2 (DE3) (Engineered)	Glucose	Yeast extract	DAP	16.11	Xu et al. (2018)
*E*. *coli* BL2 (DE3) (Engineered)	Acetaldehyde	Yeast extract, peptone	DAP	94.00	Peng et al. (2020a)
*Lactococcus lactis* subsp. *lactis* biovar. *diacetylactis* FC1	Galactose	Arginine	ND	0.81	Kim et al. (1994)
*Monacus* strain M-3	Starch	Peptone	ND	13.49^c^	Jia et al. (2017a)
*Paenibacillus polymyxa* CICC 23617	Glucose, acetoin	Yeast extract	DAP	14.90	Xiao et al. (2014)
*P*. *polymyxa* CICC 23617	Glucose	Yeast extract	DAP	3.52	Xiao et al. (2014)
*Serratia marcescens* CICC 10187	Sucrose, corn steep liquor powder, acetoin	Ammonium citrate, corn steep liquor powder	DAP	18.97	Xiao et al. (2014)
*Saccharomyces Cerevisiae* (Engineered)	Corn flour	Corn flour	ND	10.55^b^	Cui et al. (2020)

a ND, no data

b g/kg dry substrate

c mg/L.

**FIGURE 1:**
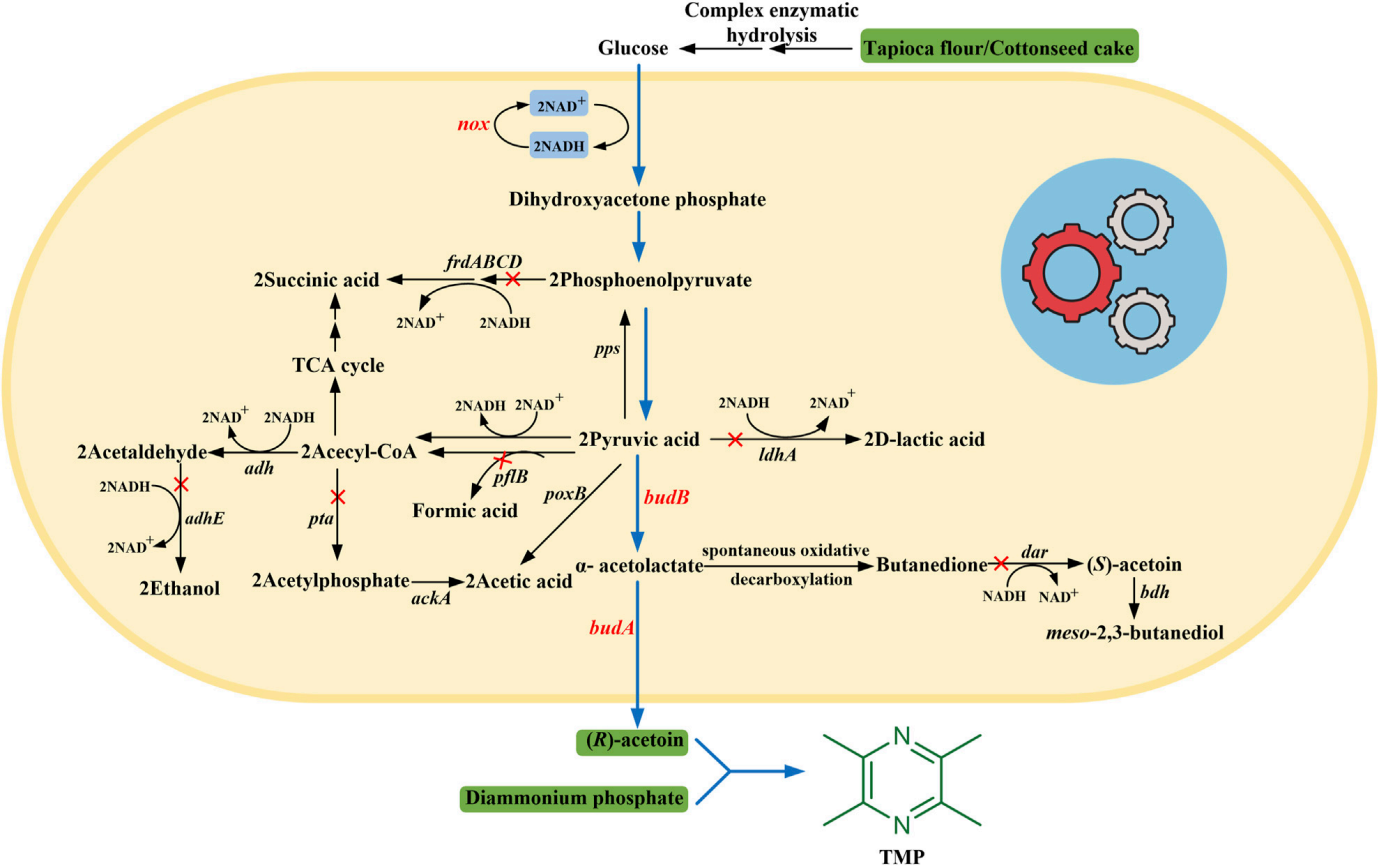
The engineered metabolic pathway for (*R*)-AC synthesis in *E*. *coli*. The *blue arrows* indicate the engineered synthesis pathway of (*R*)-AC. The *red cross marks* indicate the disrupted pathways. *budA* and *budB*, the genes encoding α-acetolactate decarboxylase and *α*-acetolactate synthase from *Enterobacter cloacae*, respectively; *nox*, the gene encoding NADH oxidase from *Lactobacillus brevis*; *dar*, diacetyl reductase gene; *frdABCD*, fumarate reductase gene; *pflB*, pyruvate formate lyase gene; *ldhA*, lactate dehydrogenase gene; *pta*, phosphotransacetylase gene; *adhE*, alcohol dehydrogenase gene.

In this study, we proposed a biosynthetic strategy to enable (*R*)-AC production in microbial cell factories from inexpensive non-food feedstocks and to achieve high-yield production of TMP in a high-pressure microreactor. In detail, we first constructed a whole-cell biocatalytic system for (*R*)-AC biosynthesis from glucose. To improve (*R*)-AC production, an in situ-NAD^+^ regeneration system was introduced into recombinant strains to balance the NADH/NAD^+^ redox. Alongside this, the biosynthetic pathways of major by-products, which are unfavorable for cell growth and the production of target products, were blocked or impaired using metabolic engineering strategies (Figure 1). Then, the hydrolysates of various non-food raw materials, which are abundantly available, were used to replace expensive carbon and nitrogen sources. The fermentation performances of different non-food feedstocks were compared. Finally, by mixing the fermentation broth with diammonium phosphate [DAP, (NH_4_)_2_HPO_4_] in a high-pressure microreactor under suitable reaction conditions, the efficient synthesis of TMP with a high titer at 56.72 g/L was achieved.

## 2 Materials and Methods

### 2.1 Chemicals

TMP (98%) was purchased from Aladdin Biochemical Technology Co., Ltd. (Shanghai, China). AC (>98%) was obtained from Tokyo Chemical Industry Co., Ltd. (Shanghai, China). Acetonitrile (HPLC grade) was purchased from Fisher Scientific. Ampicillin was obtained from Solarbio Life Sciences (Beijing, China). Tapioca flour and cassava starch were provided by Guangxi State Farms Mingyang Biochemical Group, Inc. (Nanning, China). Potato starch and corn starch were purchased from Shanghai Kaiyang Biotechnology Co., Ltd. (Shanghai, China). Cane molasses was obtained from Guangxi Sugar Industry Group (Nanning, China). Cottonseed cake was obtained from Jinan Oumi Biotechnology Co., Ltd. (Jinan, China). Other raw nitrogen sources were purchased from Kerui Biotechnology Co., Ltd. (Qingdao, China). Thermostable α-amylase (Liquozyme Supra, 60 KNU/ml) and glucoamylase (Dextrozyme, 350 AGU/ml) obtained from Novozymes (Novozymes China Biotechnology Co., Ltd.) were used for liquefaction and saccharification of raw starch. The acid protease (200 KU/g) was purchased from Imperial Jade Biotechnology Co., Ltd. (Ningxia, China) for protein hydrolysis. All other chemicals used in this study were of analytical grade and commercially available. All aqueous solutions were prepared with ultrapure water (18.2 MΩcm, PURELAB Classic, ELGA).

### 2.2 Bacterial Strains and Plasmids

All bacterial strains and plasmids used in this study are listed in Table 2. All engineered *E*. *coli* strains were constructed from MG1655 for the biosynthesis of AC. The following genes were used in this study: *budA*, *α*-acetolactate decarboxylase (GenBank accession number CP003678.1); *budB*, *α*-acetolactate synthase (GenBank accession number CP003678.1); *alsD*, *α*-acetolactate decarboxylase (GenBank accession number MK508992.1); *alsS*, *α*-acetolactate synthase (GenBank accession number MK508991.1); *nox*, NADH oxidase (GenBank accession number AF536177.1). The codons of the above genes were optimized, and the ribosomal binding site and spacer sequence containing the nucleotide sequence taaggaggatataca were added in front of each gene and then linked into gene clusters of *budB*-*budA*-*nox* and *alsS*-*alsD*-*nox*, respectively, which were artificially synthesized by Suzhou Jinweizhi Biotechnology Co., Ltd. (Suzhou, China). To enable the production of AC and balance the cellular NADH/NAD^+^ ratios in *E*. *coli*, expression plasmids of pTrc99A-*budB*-*budA*-*nox* and pTrc99A-*alsS*-*alsD*-*nox* were constructed and transformed into competent cells, respectively. Several mutant *E*. *coli* strains were constructed by stepwise deletions of genes (*dar*, *frdABCD*, *pflB*, *ldhA*, *pta* and *adhE*) encoding key enzymes in the metabolic pathways via homologous recombination to reduce by-products. The effects of metabolic pathway optimization on glucose consumption, fermentation efficiency, and finial concentrations of AC and by-products were investigated to screen high-yield recombinant strains.

**TABLE 2:** Bacterial strains and plasmids used in this study.

Strains and plasmids	Detailed information	Source
*E*. *coli* MG1655	Parent strain	Lab collection
GXASR	*E*. *coli* MG1655/pTrc99A	This study
GXASR1	*E*. *coli* MG1655/pTrc99A-*budB*-*budA*-*nox*	This study
GXASR2	*E*. *coli* MG1655/pTrc99A-*alsS*-*alsD*-*nox*	This study
GXASR3	*E*. *coli* MG1655 Δ*dar*/pTrc99A-*budB*-*budA*-*nox*	This study
GXASR4	*E*. *coli* MG1655 Δ*frdABCD*/pTrc99A-*budB*-*budA*-*nox*	This study
GXASR5	*E*. *coli* MG1655 Δ*dar* Δ*frdABCD*/pTrc99A-*budB*-*budA*-*nox*	This study
GXASR6	*E*. *coli* MG1655 Δ*dar* Δ*frdABCD* Δ*pflB*/pTrc99A-*budB*-*budA*-*nox*	This study
GXASR7	*E*. *coli* MG1655 Δ*dar* Δ*frdABCD* Δ*ldhA*/pTrc99A-*budB*-*budA*-*nox*	This study
GXASR8	*E*. *coli* MG1655 Δ*dar* Δ*frdABCD* Δ*pta*/pTrc99A-*budB*-*budA*-*nox*	This study
GXASR9	*E*. *coli* MG1655 Δ*dar* Δ*frdABCD* Δ*adhE*/pTrc99A-*budB*-*budA*-*nox*	This study
GXASR10	*E*. *coli* MG1655 Δ*dar* Δ*frdABCD* Δ*pflB* Δ*ldhA*/pTrc99A-*budB*-*budA*-*nox*	This study
GXASR11	*E*. *coli* MG1655 Δ*dar* Δ*frdABCD* Δ*pflB* Δ*ldhA* Δ*pta*/pTrc99A-*budB*-*budA*-*nox*	This study
pTrc99A	Amp^r^	Lab collection
pTrc99A-*budB*-*budA*-*nox*	Amp^r^	This study
pTrc99A-*alsS*-*alsD*-*nox*	Amp^r^	This study

alsD and alsS, the genes encoding α-acetolactate decarboxylase and α-acetolactate synthase from P. polymyxa, respectively.

### 2.3 Culture Medium and Growth Conditions

The Luria-Bertani (LB) medium (10.0 g/L tryptone, 5.0 g/L yeast extract, and 10.0 g/L NaCl) at pH 7.0 was used for routine culture of *E*. *coli* (Xu et al., 2018). The initial fermentation medium, which contained 100.0 g/L glucose, 10.0 g/L peptone, 7.0 g/L yeast extract, 0.5 g/L NaCl, 0.2 g/L MgSO_4_, 0.5 g/L betaine monohydrate and 0.1 g/L thiamine, was used for (*R*)-AC production by recombinants. The pH was adjusted to 7.0 before sterilization. Glucose solution was sterilized separately then added into the autoclaved initial fermentation medium to make a final concentration of 100 g/L before inoculation. Ampicillin was added into the medium as required with a final concentration of 0.1 g/L. Five percent (v/v) of the inoculant was used for all fermentation processes unless indicated otherwise. All strains were incubated in 250 ml Erlenmeyer flasks with maximally 50 ml medium at 37°C on a rotary shaker at 250 rpm. Cell growth was determined via OD_600_ (optical density at 600 nm) using a DU 800 spectrophotometer (Beckman Coulter). All the experiments in shake flasks were performed in triplicate.

### 2.4 Preparation of Non-Food Medium

Instead of commercial carbon and nitrogen sources, the non-food medium containing fermentable sugar and nitrogen derived from raw materials by enzymatic hydrolysis was prepared in three consecutive steps: pre-treatment, liquefaction, and saccharification-protein hydrolysis. In the pre-treatment step, 56.4 g tapioca flour and 20.1 g cottonseed cake were added into a 500 ml Erlenmeyer flask with 300 ml tap water. The pH was adjusted to 6.3 using 20% NaOH solution and 7.5 ml *α*-amylase solution (0.5 g CaCl_2_ was added to 2 ml *α*-amylase, then the final volume was adjusted to 100 ml with distilled water) was added before autoclaving at 121°C for 15 min to degrade the structure of starch grains for better hydrolysis performance. In the subsequent liquefaction step, the pH was adjusted to 6.3 and 15 ml *α*-amylase solution was added. The hydrolysate was incubated on a water bath shaker (95°C, 160 rpm) for 1 h and allowed to cool. In the last step, the hydrolysate was adjusted to pH 4.3 using 20% H_2_SO_4_ solution and then treated with 22.5 ml glucoamylase solution (4 ml glucoamylase was diluted to 100 ml with distilled water) and 0.6 g acid protease on a water bath shaker (55°C, 160 rpm) for 20 h. Afterwards, the pH was readjusted to 7.0 using 20% NaOH solution and the supernatant was collected by centrifugation at 6,000 rpm for 5 min. The final non-food medium was sterilized at 115°C for 15 min.

### 2.5 Optimization of Fermentation Parameters for AC Synthesis

The optimal fermentation conditions were identified respectively in shake flasks and 1-L fermenters according to the titer of (*R*)-AC. In shake-flask fermentation, four fermentation parameters (temperature, fermentation volume, inoculation proportion and rotate speed) were investigated. Fermentation conditions, including the initial pH, aeration rate and agitation speed, were optimized in a multiple parallel fermenter system (1-L working volume, BLBIO-1GC-5-H, Shanghai Bailun Bio-Technology Co., Ltd.) with an initial broth volume of 0.5 L. During the entire fermentation, the concentrations of residual glucose and (*R*)-AC were examined regularly. The fermentation process was terminated when the residual glucose decreased below 5 g/L.

### 2.6 Fed-Batch Fermentation

Based on the optimal condition determined from preliminary work, a 5-L fermenter (BIOTECH-5BG, Shanghai Baoxing Bio-Engineering Equipment Co., Ltd.) was employed to achieve higher (*R*)-AC production via fed-batch fermentation. When the residual glucose in the fermentation broth fell below 40 g/L, the concentrated hydrolysates of tapioca flour and cottonseed cake by rotary evaporation were supplemented to make the glucose to approximately 140 g/L. The fermentation process was terminated when the residual glucose declined to 10 g/L or when the titer of (*R*)-AC stopped increasing. The concentrations of (*R*)-AC and major by-products were analyzed using gas chromatography (GC) and high-performance liquid chromatography (HPLC), respectively.

### 2.7 Synthesis of 2,3,5,6-Tetramethylpyrazine

An appropriate ammonium salt is crucial to the synthesis of TMP. Previous studies have revealed that DAP was the most efficient ammonium salt to support TMP synthesis among various ammonium sources (Zhu and Xu 2010b; Zhong et al., 2020a). The reaction system in this study was a mixture of fermentation broth and DAP at a total volume of 20 ml. All condensation reactions were carried out in a high-pressure microreactor (LHK-25, OLEBVAP Instrument and Equipment Co., Ltd., China). To improve the efficiency of TMP production, reaction parameters including temperature, rotation speed, reaction time, the molar ratio of AC/DAP and the initial pH were optimized. Due to the poor water solubility of TMP, ethanol was added into the reaction mixture to reach a volume content of 60% after the condensation reaction (Xu et al., 2018). After ultrasonication for 5 min, the reaction solution was centrifuged at 12,000 rpm for 2 min to resolve the TMP into ethanol. The solution was filtered, then immediately subjected to GC analysis after an appropriate dilution.

### 2.8 Analytical Methods

The fermentation broth was centrifuged at 12,000 rpm for 2 min, then the supernatant was collected and diluted for the determination of residual glucose concentration using a biosensor analyzer (SBA-40D, Shandong, China). The nitrogen content in the raw materials was calculated according to the production instructions.

The concentrations of AC and TMP were determined by a GC-FID system (Agilent 7890A) equipped with a polar column (Phenomenex ZB-WAX Plus, 30 m × 0.32 mm × 0.25 μm) with nitrogen as carrier gas at a constant flow of 1.6 ml/min. The column oven temperature was maintained at 100°C for 1 min, 20°C/min ramp until 180°C, and held at 180°C for 3 min. The injector and detector temperatures were both at 250°C. The concentrations of AC and TMP were calculated by the internal standard method using acetonitrile as an internal standard.

The concentrations of (*R*)-AC and (*S*)-AC in the fermentation broth were analyzed as described in our previous study (Li et al., 2018). Briefly, (*R*)-AC and (*S*)-AC in the supernatant of fermentation broth were extracted with ethyl acetate and differentiated by the GC system (Agilent 7890A) equipped with a chiral column (Agilent CycloSil-B, 0.32 mm × 0.25 mm×30 m). The optical purity of (*R*)-AC was calculated as follows: (*R*)/[(*S*)+(*R*)]×100%, where (*R*) and (*S*) represented the concentrations of (*R*)-AC and (*S*)-AC, respectively.

The fermentation broth was centrifuged at 13,000 rpm for 5 min and the supernatant was filtered through a 0.2-μm nylon syringe filter. The main by-products, including acetate, succinate, formate and lactate, were determined using HPLC equipped with a Carbomix H-NP5 column (8%, 7.8 × 300 mm, Sepax Technologies) at 55°C. The analysis was conducted with a mobile phase of 2.5 mM H_2_SO_4_ at a flow rate of 0.6 ml/min and the UV detection wavelength was 210 nm. The injection volume of each sample was 10 μL. The by-product 2,3-butanediol (2,3-BD) was determined by a GC-FID system as mentioned above.

## 3 Results and Discussion

### 3.1 Screening of Suitable Genes Derived From Different Microbial Sources for AC Production

The synthesis of TMP by microorganisms, including *Bacillus* sp., (Xiao et al., 2006), *Corynebacterium* sp. (Dickschat et al., 2010), *Lactococcus* sp. (Kim et al., 1994), *Chondromyces* sp. (Dickschat et al., 2005), *Streptomyces* sp. (Braña et al., 2014) and *Monacus* sp. (Jia et al., 2017a), has been previously reported. However, the maximum production of TMP from the above microorganisms was only 4.33 g/L, which significantly hindered the industrial application. Regarding the synthesis mechanism of TMP *in vivo*, there exist two conflicting viewpoints. Compared with a standpoint of an enzymatic condensation process, the predominant experimental results tend to support the viewpoint that the precursor of TMP was biosynthesized *in vivo* whereas the subsequent condensation was an *in vitro* nonenzymatic process (Zhu et al., 2010; Xiao et al., 2014). Hence, it was essential to investigate the biosynthesis process of AC, which was the limiting step for TMP synthesis, and further tackle the problem of low AC production.

Biosynthesis of AC by endogenous strains has been widely reported (Romano and Suzzi, 1996; Cui et al., 2020; Lu et al., 2020; Zhong et al., 2020b). Nevertheless, wild-type strains often suffered from low capability in AC generation (Xiao et al., 2010; Xu et al., 2015; Guo et al., 2017). Fortunately, the synthesis of AC directed by genes from different bacteria species could be achieved in heterologous hosts by genetic engineering approaches (Yamada-Onodera et al., 2002), therefore the selection of suitable non-native producers is of great importance. Amongst engineered microbial strains for AC biosynthesis, *E*. *coli* is frequently used as a desirable candidate due to a comprehensive understanding of the metabolisms and well-established genetic engineering tools (Adamczyk and Reed 2017). The *α*-acetolactate synthase and *α*-acetolactate decarboxylase are two crucial enzymes involved in AC biosynthetic pathway (Celińska and Grajek 2009; Ji et al., 2011; Jang et al., 2017). Because of the compatibility with the chassis cells, the selection of suitable genes from proper donors is one of the key issues (Xiao and Lu 2014). Additionally, as shown in Figure 1, excess NADH would be generated during the production of (*R*)-AC by *E*. *coli*. To balance the *in vivo* NADH/NAD^+^ redox, the bypasses of organic acids and alcohols must be activated to consume the surplus NADH. By the expression of exogenous NADH oxidase, the synthesis of by-products could be diminished, consequently leading to the redistribution of carbon flux for (*R*)-AC (Sun et al., 2012; Zhang et al., 2014).

**FIGURE 2:**
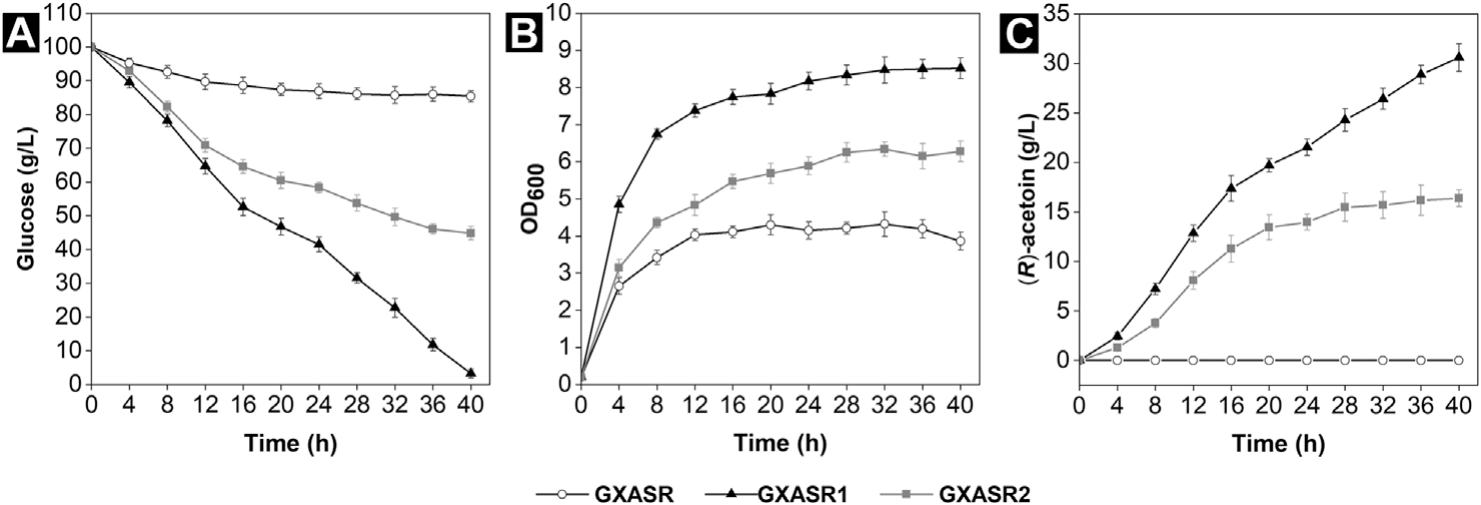
Synthesis of (*R*)-AC by GXASR (pTrc99A) and genetically engineered strains harboring different exogenous genes using 50 ml initial fermentation medium at pH 7.0 in 250 ml shake flasks at 250 rpm and 37°C for 40 h **(A)** residual glucose **(B)** cell growth **(C)** (*R*)-AC production. Error bars represent the standard deviations.

Here, we compared the AC production efficiencies of two recombinants, which carried different genes encoding *α*-acetolactate decarboxylase and *α*-acetolactate synthase from *E*. *cloacae* and *P*. *polymyxa*, respectively. Meanwhile, the gene of NADH oxidase from *L*. *brevis* was introduced into the recombinants to construct an endogenous cofactor regeneration system. Figure 2C showed that the strain GXASR (pTrc99A) was incapable of producing (*R*)-AC during fermentation. In contrast, the (*R*)-AC production of strain GXASR1 (pTrc99A-*budB*-*budA*-*nox*) achieved 30.59 g/L, which represented a 1.87-fold increase compared to strain GXASR2 (pTrc99A-*alsS*-*alsD*-*nox*) (16.37 g/L). These results demonstrated that the exogenous genes derived from both *E*. *cloacae* and *P*. *polymyxa* were effectively expressed in *E*. *coli*, enabling efficient (*R*)-AC production. Moreover, the accumulation of (*R*)-AC, cell growth and glucose-consuming ability of strain GXASR1 were all far surpassing that of strain GXASR2. This was because the activities of enzymes from different gene sources could vary widely (Nadal et al., 2009; Xu et al., 2015). In our case, genes of *budB* and *budA* from *E*. *cloacae* could probably express *α*-acetolactate synthase and *α*-acetolactate decarboxylase with higher activities. A similar result was observed by Xu et al. (2018) when comparing the AC production from recombinants carrying different exogenous genes, and they speculated that it was because *Enterobacter* had a close codon preference with *E*. *coli* and both of them belonged to the Enterobacteriaceae.

### 3.2 Effect of Inducer Concentrations

As the improvement of the enzymatic activities and protein expression efficiency could enhance the AC production, we further examined the effect of inducer concentrations on (*R*)-AC production for strain GXASR1. It was essential to optimize the concentration of isopropyl-*β*-D-thiogalactopyranoside (IPTG) because protein expression was not positively related to IPTG concentration (Couto et al., 2017). When OD_600_ reached approximately 2.0, different concentrations (0–1.00 mM) of IPTG were evaluated for induction. As shown in Table 3, the production of (*R*)-AC attained 30.59 g/L without induction, whereas a higher titer of (*R*)-AC at 32.70 g/L was detected in the presence of 0.01 mM IPTG. The pTrc99A plasmid is a constitutive expression vector, while the *trc* promoter is also inducible by IPTG, which is an analogue of lactose binding specifically to the repressor protein of the *lac* operon. The addition of IPTG into the medium could make up for the insufficiency of *lac* inducer expressed by the pTrc99A plasmid, resulting in higher expression of plasmid and therefore enhanced the production of (*R*)-AC. However, both the cell growth and the (*R*)-AC synthesis ability of strain GXASR1 gradually decreased along with the increase of IPTG concentration. Considering that IPTG was not an innocuous inducer, this result indicated that there was a toxic effect of excessive IPTG on the cell proliferation as well as (*R*)-AC productivity of GXASR1. Therefore, a final concentration of IPTG at 0.01 mM was the most desirable for recombinant cell induction and was adopted in the following experiments.

**TABLE 3:** Fermentation performances (mean ± SD) of *E*. *coli* GXASR1 under different IPTG induced concentrations in 40 h.

Strain	IPTG (mM)	Glucose consumed (g/L)	OD_600_	(*R*)-AC (g/L)	Yield (g/g)^a^	Productivity [g/(L·h)^b^]
GXASR1	0	96.8 ± 0.6	8.52 ± 0.18	30.59 ± 1.15	0.32	0.76
	0.01	97.3 ± 1.3	8.48 ± 0.37	32.70 ± 0.68	0.34	0.82
	0.05	95.1 ± 2.1	8.26 ± 0.49	28.64 ± 0.93	0.30	0.72
	0.10	93.6 ± 1.6	7.96 ± 0.51	27.38 ± 0.55	0.29	0.68
	1.00	86.7 ± 2.3	6.8 ± 0.35	22.44 ± 0.63	0.26	0.56

a Production of AC/glucose consumption.

b Production of AC/fermentation time; SD, standard deviation, n = 3.

### 3.3 Optimization of Metabolic Pathways in Recombinant Strains

As illustrated in Figure 1, a variety of by-products were produced along with the biosynthesis of (*R*)-AC, which led to a decrease of carbon flux in the synthetic pathway of (*R*)-AC and therefore restricted its production. Our results showed that (*R*)-AC produced by GXASR1 reached a titer of 32.70 g/L with an optical purity of 99.14%, while the main by-products were determined as 3.37 g/L 2,3-BD, 2.13 g/L acetate, 0.63 g/L lactate, 0.41 g/L formate, 0.37 g/L ethanol and 1.44 g/L succinate. Genetic manipulation on critical metabolic nodes has been widely used to reconstruct the metabolic network of microbial cells. It has been reported that different genes encoding pathway enzymes, which catalyzed the formation of diverse by-products, were deleted from the microbial genome to facilitate the AC production (Förster et al., 2017; Jang et al., 2017). Consequently, after analyzing the metabolic pathway of the host bacteria we performed genetic modification to block the biosynthetic pathways of major by-products which were not essential for cell growth. In detail, we first deleted the diacetyl reductase gene (*dar*) to decrease the flux from *α*-acetolactate to *meso*-2,3-BD via (*S*)-AC (Figure 1). After *dar* gene deletion, 2,3-BD was still produced by GXASR3, but its concentration markedly dropped by 57.27% while the production of (*R*)-AC slightly increased from 32.70 to 34.65 g/L (Table 4). Parallelly, we deleted the fumarate reductase gene (*frdABCD*) to impair the synthesis of succinate, resulting in an 11.44% increase of (*R*)-AC in GXASR4 (Δ*frdABCD*) compared to that in GXASR1. The concentrations of 2,3-BD and succinate from the double knockout GXASR5 (Δ*dar* Δ*frdABCD*) dramatically decreased 63.20 and 83.33% compared to that from GXASR1, respectively. Compared with GXASR1, the strain of GXASR5 (Δ*dar* Δ*frdABCD*) showed a 63.20% decrease in 2,3-BD and an 83.33% decrease in succinate. Besides, the output of (*R*)-AC improved about 13.94% with a higher enantiomeric purity.

**TABLE 4:** Fermentation performance (mean ± SD) of genes knockout engineered strains in shake flasks using initial fermentation medium.

Strains	Glucose consumed (g/L)	(*R*)-AC (g/L)	(*S*)-AC (g/L)	2,3-BD (g/L)	Acetate (g/L)	Lactate (g/L)	Formate (g/L)	Ethanol (g/L)	Succinate (g/L)	Yield (g/g)	Productivity of (*R*)-AC [g/(L·h)]	Purity of (*R*)-AC (%)
GXASR1	97.3 ± 1.5	32.70 ± 0.44	0.27 ± 0.07	3.37 ± 0.28	2.13 ± 0.08	0.63 ± 0.07	0.41 ± 0.05	0.37 ± 0.05	1.44 ± 0.19	0.34	0.82	99.14
GXASR3	96.5 ± 0.8	34.65 ± 1.23	0.23 ± 0.04	1.44 ± 0.15	1.98 ± 0.12	0.79 ± 0.07	0.50 ± 0.04	0.44 ± 0.07	1.51 ± 0.10	0.36	0.87	99.35
GXASR4	94.3 ± 1.1	36.44 ± 0.78	0.27 ± 0.04	1.35 ± 0.07	2.07 ± 0.17	0.84 ± 0.11	0.46 ± 0.04	0.50 ± 0.10	0.28 ± 0.04	0.39	0.91	99.26
GXASR5	97.4 ± 1.6	37.26 ± 1.06	0.27 ± 0.09	1.24 ± 0.14	2.03 ± 0.08	0.69 ± 0.10	0.52 ± 0.03	0.41 ± 0.04	0.24 ± 0.02	0.38	0.93	99.28
GXASR6	94.5 ± 1.0	39.18 ± 0.85	0.22 ± 0.09	0.89 ± 0.22	1.84 ± 0.11	0.62 ± 0.11	0.00	0.38 ± 0.04	0.30 ± 0.00	0.41	0.98	99.45
GXASR7	95.6 ± 0.8	38.39 ± 0.84	0.26 ± 0.07	1.04 ± 0.18	1.67 ± 0.09	0.24 ± 0.02	0.38 ± 0.03	0.62 ± 0.08	0.16 ± 0.00	0.40	0.96	99.32
GXASR8	96.2 ± 0.8	38.57 ± 1.92	0.25 ± 0.01	0.96 ± 0.10	0.57 ± 0.06	0.49 ± 0.05	0.30 ± 0.00	0.39 ± 0.04	0.20 ± 0.03	0.40	0.96	99.35
GXASR9	50.2 ± 3.2	19.74 ± 1.17	0.14 ± 0.03	0.17 ± 0.05	4.33 ± 0.19	0.21 ± 0.02	1.28 ± 0.03	0.00	0.00	0.39	0.49	99.28
GXASR10	95.4 ± 1.8	39.55 ± 0.86	0.25 ± 0.05	1.16 ± 0.11	1.63 ± 0.11	0.15 ± 0.04	0.00	0.50 ± 0.10	0.28 ± 0.08	0.41	0.99	99.36
GXASR11	93.4 ± 0.6	40.84 ± 1.15	0.24 ± 0.05	1.26 ± 0.14	0.37 ± 0.04	0.18 ± 0.02	0.00	0.41 ± 0.03	0.14 ± 0.04	0.44	1.02	99.42

SD, standard deviation, n = 3.

Based on the double knockout strain GXASR5, the genes of *pflB*, *ldhA*, *pta* and *adhE* were respectively deleted to construct triple knockouts of GXASR6 (Δ*dar* Δ*frdABCD* Δ*pflB*), GXASR7 (Δ*dar* Δ*frdABCD* Δ*ldhA*), GXASR8 (Δ*dar* Δ*frdABCD* Δ*pta*) and GXASR9 (Δ*dar* Δ*frdABCD* Δ*adhE*). The synthesis pathway of formate was successfully blocked in GXASR6 (Δ*dar* Δ*frdABCD* Δ*pflB*) since no formate was detected during fermentation. The production of lactate declined from 0.69 g/L in GXASR5 to 0.24 g/L in GXASR7, while the concentration of acetate in GXASR8 was reduced by 73.24%. Additionally, the productions of (*R*)-AC in GXASR6, GXASR7 and GXASR8 were slightly higher than that of GXASR5. The results demonstrated that further deletion of *pflB*, *ldhA* and *pta* positively affected the accumulation of AC.

The mutant GXASR9 devoid of *adhE* was unable to produce ethanol. However, as the glucose consumption was significantly reduced, the AC output declined to 19.74 g/L, approximately 50% of that in GXASR5. This observation was in agreement with other studies (Li et al., 2015a; Förster et al., 2017), indicating that deletion of *adhE* negatively affected the cell growth and metabolism, and eventually led to a significant decrease of AC. Therefore, instead of deleting *adhE*, the quadruple and quintuple knockouts of GXASR10 (Δ*dar* Δ*frdABCD* Δ*pflB* Δ*ldhA*) and GXASR11 (Δ*dar* Δ*frdABCD* Δ*pflB* Δ*ldhA* Δ*pta*) were further constructed respectively. Among all the recombinants, GXASR11 yielded the highest level of (*R*)-AC at 40.84 g/L with an enantiomeric purity of 99.42% (Table 4), which was higher than that obtained (97.3%) by Xu et al. (2015) using metabolic engineered *E. coli* involving the *bud*AB genes for (*R*)-AC biosynthesis. Although a small amount of ethanol was accumulated by this deletion mutant, the concentrations of other by-products obviously descended while no formate was produced, indicating that the deletions of *dar*, *frdABCD*, *pflB*, *ldhA* and *pta* were beneficial for the synthesis of (*R*)-AC. As a result, GXASR11 was chosen as the best AC producer for the subsequent fermentation experiments in shake flasks and fermenters.

### 3.4 Screening for Optimal Carbon Source and Concentration

To provide an eco-friendly and economical bio-based alternative for AC and TMP production, not only the yield and productivity of the biosynthetic process should be optimized for precursor production, but also the cost of manufacture, particularly fermentation medium costs, needs to be carefully evaluated. Glucose is a common carbon source used in laboratory-scale cultivation processes for heterotrophic microorganisms. As the cost of raw materials significantly contributes to the manufacturing cost of target products, refined glucose is not desirable for practical production due to its relatively high price. Therefore, in place of using glucose as the sole carbon source, here we employed several cheap and abundant feedstocks for (*R*)-AC biosynthesis aiming to reduce the overall manufacturing cost of TMP.

**FIGURE 3:**
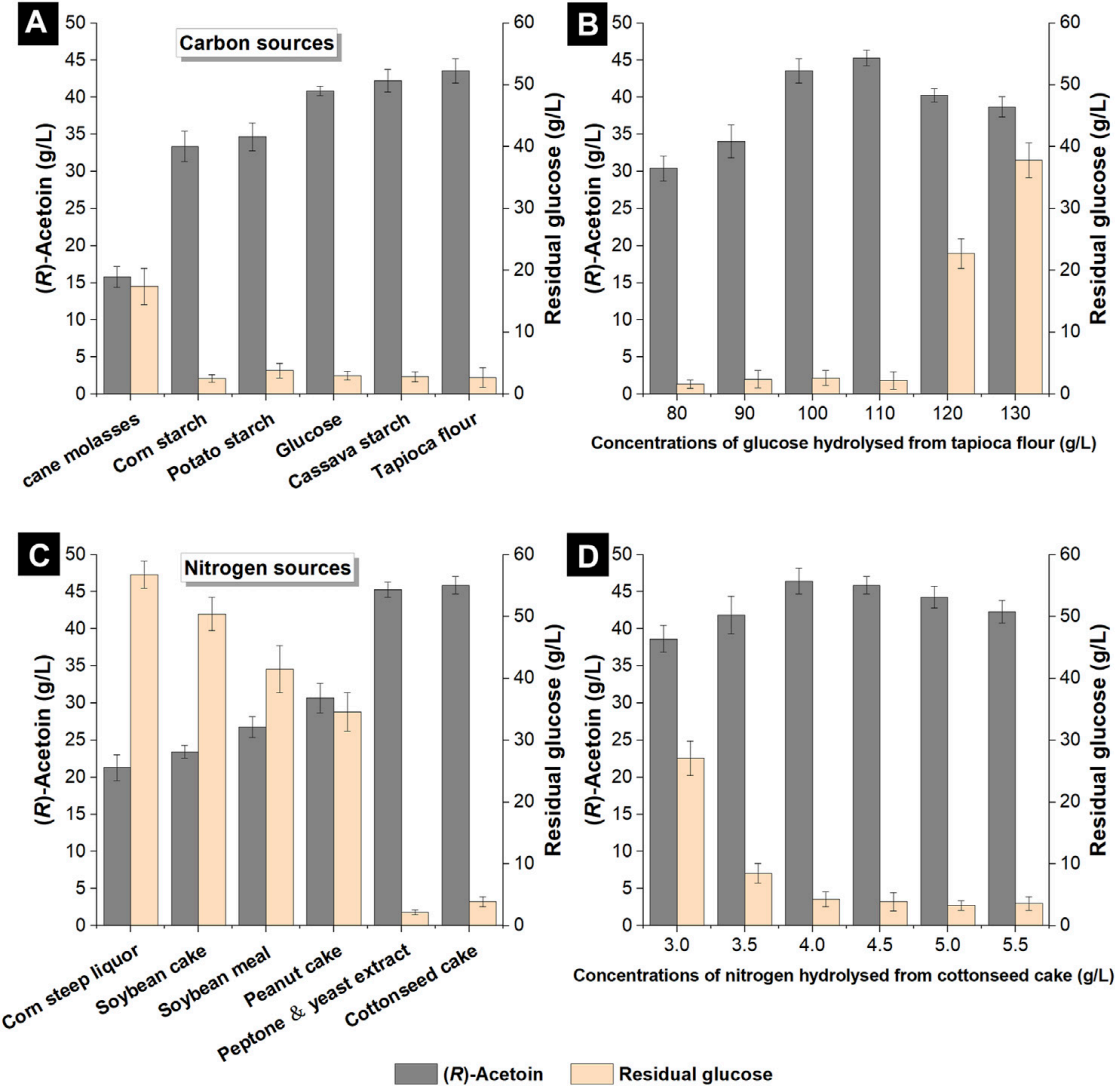
Effects of different carbon and nitrogen sources from hydrolysates of cheap raw materials on the production of (*R*)-AC by GXASR11 in 250 ml shake flasks at 250 rpm and 37°C. **(A)** various carbon sources **(B)** effects of glucose concentrations on (*R*)-AC production **(C)** various nitrogen sources **(D)** effects of nitrogen concentrations on (*R*)-AC production.

The fermentation performances of glucose and five different non-food carbon sources (cane molasses, corn starch, potato starch, cassava starch and tapioca flour) for (*R*)-AC production by engineered *E*. *coli* were compared. Different raw materials were converted into fermentable sugar (i.e., glucose) by enzymatic hydrolysis, which was added into the non-food medium to obtain an initial glucose concentration of 100 g/L. Fermentation was carried out in shake flasks for 40 h, by the end of which the production of (*R*)-AC and the concentration of residual glucose were examined. As shown in Figure 3A, the hydrolyzed cane molasses gave the lowest (*R*)-AC production (15.81 g/L) among all carbon sources, while the residual glucose concentration was up to 17.4 g/L. Though cane molasses could be directly utilized in fermentation by yeasts for a high yield of ethanol and other chemicals (Basso et al., 2011; Abubaker et al., 2012), it could not be effectively metabolized by GXASR11. This is because the major component of cane molasses is sucrose, a kind of non-reducing sugar (Su et al., 2021), and the ability and efficiency of *E*. *coli* to consume sucrose depend on the specific strain used (Reid and Abratt 2005; Mohamed et al., 2019). The hydrolyzed corn starch and potato starch could provide glucose for fermentation (Liu 2002; Chen and Zhang 2012), however, the high viscosity of these hydrolysates led to low efficiency in mass transfer, resulting in a negative impact on cell growth and (*R*)-AC production. The outputs of (*R*)-AC from hydrolyzed cassava starch and tapioca flour reached 42.24 g/L and 43.55 g/L, respectively, both of which surpassed that from glucose (40.84 g/L). These hydrolysates were relatively less viscous than that of corn starch and potato starch, thus they did not negatively affect the mass transfer. Moreover, besides the glucose hydrolyzed from starch, amino acids and fatty acids were also released from the plant-derived constituents, serving as nutrients and growth factors for cell proliferation and resulting in more efficient (*R*)-AC synthesis. Additionally, the processing technology of tapioca flour, which only involved processes of dehydration and grinding for the peeled cassava root, was much easier and less costly than that of cassava starch, which required an extra refining process. Given that the price of tapioca flour was approximately 30–50% lower than that of cassava starch as well as its accessibility, tapioca flour was selected as the optimal alternative carbon source for (*R*)-AC fermentation.

To assess the optimal concentration of tapioca flour, its hydrolysate was prepared to obtain 80–130 g/L glucose in the non-food medium. The production of (*R*)-AC by GXASR11 was examined when the residual glucose fell below 5 g/L or remained unchanged in the fermentation broth. As shown in Figure 3B, the concentration of (*R*)-AC increased along with the increase of glucose concentration from 80 to 110 g/L, and peaked at 110 g/L glucose with an AC output of 45.28 g/L. Nevertheless, as the glucose level rose to 120 or 130 g/L, the glucose consumption of engineered strain significantly dropped, as well as the output of AC. This was probably due to the increase of osmotic pressure and viscosity of the fermentation broth, which in turn inhibited the cell growth (data not shown) and mass transfer efficiency.

### 3.5 Screening for Optimal Nitrogen Source and Concentration

The final composition of the non-food medium was determined by a combination of the optimum carbon and nitrogen sources, hence we further compared five different raw materials with the refined nitrogen source as shown in Figure 3C. Although nitrogen sources were not served as substrates for (*R*)-AC synthesis, nitrogen was an essential component of cell structure and enzymes, which therefore strongly influenced the fermentation efficiency of (*R*)-AC. The non-food medium was prepared by adding hydrolysates of raw materials to obtain 4.5 g/L total nitrogen.

Figure 3C showed that the hydrolysates of corn steep liquor, soybean cake, soybean meal and peanut cake were not beneficial nitrogen sources for (*R*)-AC production by GXASR11, because by the end of fermentation, the concentration of residual glucose was in the range between 34.6 g/L and 56.8 g/L, and the output of (*R*)-AC was between 21.28 g/L and 30.66 g/L. In contrast, the peptone and yeast extract were good nitrogen sources for (*R*)-AC biosynthesis because the titer achieved 45.28 g/L at 40 h. The hydrolyzed cottonseed cake turned out to be an equally promising nitrogen source for GXASR11 because a slightly higher output of (*R*)-AC at 45.89 g/L was achieved. As commonly used nitrogen sources for microbial cultivation, peptone and yeast extract could be directly utilized by microbial cells, whereas other cheap feedstocks should be enzymatically hydrolyzed into amino acids or smaller compounds before being metabolized by the recombinant *E*. *coli*. However, due to the economic factor (according to the price from Sigma Aldrich, yeast extract and peptone for microbiology were about $198 and $350 per kg, respectively), their usage was hampered in industrial-scale production. The differences in (*R*)-AC production of various feedstocks probably resulted from different hydrolysis efficiency by acid protease for the same weight of raw materials. We speculated that cottonseed cake was possibly hydrolyzed more sufficiently. In addition, the cottonseed cake contained small amounts of sugars and esters, which might contribute to the higher production of (*R*)-AC as well. These results proved the advantages that made cottonseed cake an excellent substitute for peptone and yeast extract for (*R*)-AC production. The concentration of nitrogen hydrolyzed from cottonseed cake was also optimized. As seen in Figure 3D, 4.0 g/L nitrogen from hydrolyzed cottonseed cake resulted in a maximal (*R*)-AC output at 46.46 g/L.

### 3.6 Optimization of Raw Material Hydrolysis Process for (*R*)-AC Production

The hydrolysis process of raw materials is of great significance to better utilization of low-value feedstocks for efficient AC production (Xiao et al., 2007; Zhong et al., 2020b). To acquire the optimum hydrolysis procedure, the effects of enzyme dose and hydrolysis time were investigated by single-factor experiments. As observed from Table 5, the tapioca flour was hydrolyzed by different dosages of *α*-amylase and glucoamylase for different time (8, 12, 16, 20 and 24 h). The *α*-amylase could hydrolyze the *α*-1,4 linkages in tapioca flour to produce dextrin, oligosaccharides and monosaccharides (Lévêque et al., 2000). This enzymatic processing of starch allowed a rapid reduction in the viscosity of the hydrolysate. The partially hydrolyzed starch by *α*-amylase was further treated with glucoamylase, which further hydrolyzed the chemical bonds of *α*-1,4 and *α*-1,6 to produce glucose (Ruiz et al., 2011). On the other hand, the cottonseed cake was hydrolyzed by the acid protease to generate amino acids before being utilized by *E*. *coli* as nitrogen sources. It was subjected to the acid protease at different enzyme/substrate ratios of 1, 2, 3, 4 and 5 KU/g, respectively. Given the factors including the efficiency of hydrolysis, economic feasibility and processing period, the optimum hydrolysis condition was set as follows: *α*-amylase 0.5 KNU/g, glucoamylase 5 AGU/g, acid protease 3 KU/g and 20 h for hydrolysis.

**TABLE 5:** Effects of enzymatic hydrolysis conditions on the production of (*R*)-AC (mean ± SD) in shake flasks.

Enzymatic hydrolysis conditions	(*R*)-AC (g/L)	Residual glucose (g/L)
Alpha-amylase (KNU/g)		
0.1	28.44 ± 0.83	38.8 ± 2.2
0.3	37.63 ± 1.57	29.2 ± 1.8
0.5	46.59 ± 2.04	3.8 ± 1.6
0.7	46.46 ± 1.78	4.3 ± 1.2
0.9	46.72 ± 1.44	3.5 ± 1.8
Glucoamylase (AGU/g)		
1	35.66 ± 1.81	26.6 ± 2.6
3	41.25 ± 1.65	20.1 ± 1.2
5	46.70 ± 1.93	4.6 ± 0.9
7	46.59 ± 2.04	3.8 ± 1.6
9	46.68 ± 1.26	4.3 ± 1.5
Acid protease (KU/g)		
1	37.36 ± 1.04	16.9 ± 1.5
2	46.70 ± 1.93	4.6 ± 0.9
3	48.27 ± 2.11	3.7 ± 1.2
4	48.32 ± 1.85	2.9 ± 0.9
5	48.22 ± 1.69	3.4 ± 1.1
Saccharification time (h)		
8	30.53 ± 1.26	43.6 ± 2.3
12	39.64 ± 0.86	25.7 ± 1.6
16	44.26 ± 1.62	14.6 ± 0.9
20	48.27 ± 2.11	3.7 ± 1.2
24	48.35 ± 1.88	2.8 ± 0.6

SD, standard deviation, n = 3.

### 3.7 Optimization of Fermentation Condition in Shake Flasks

**FIGURE 4:**
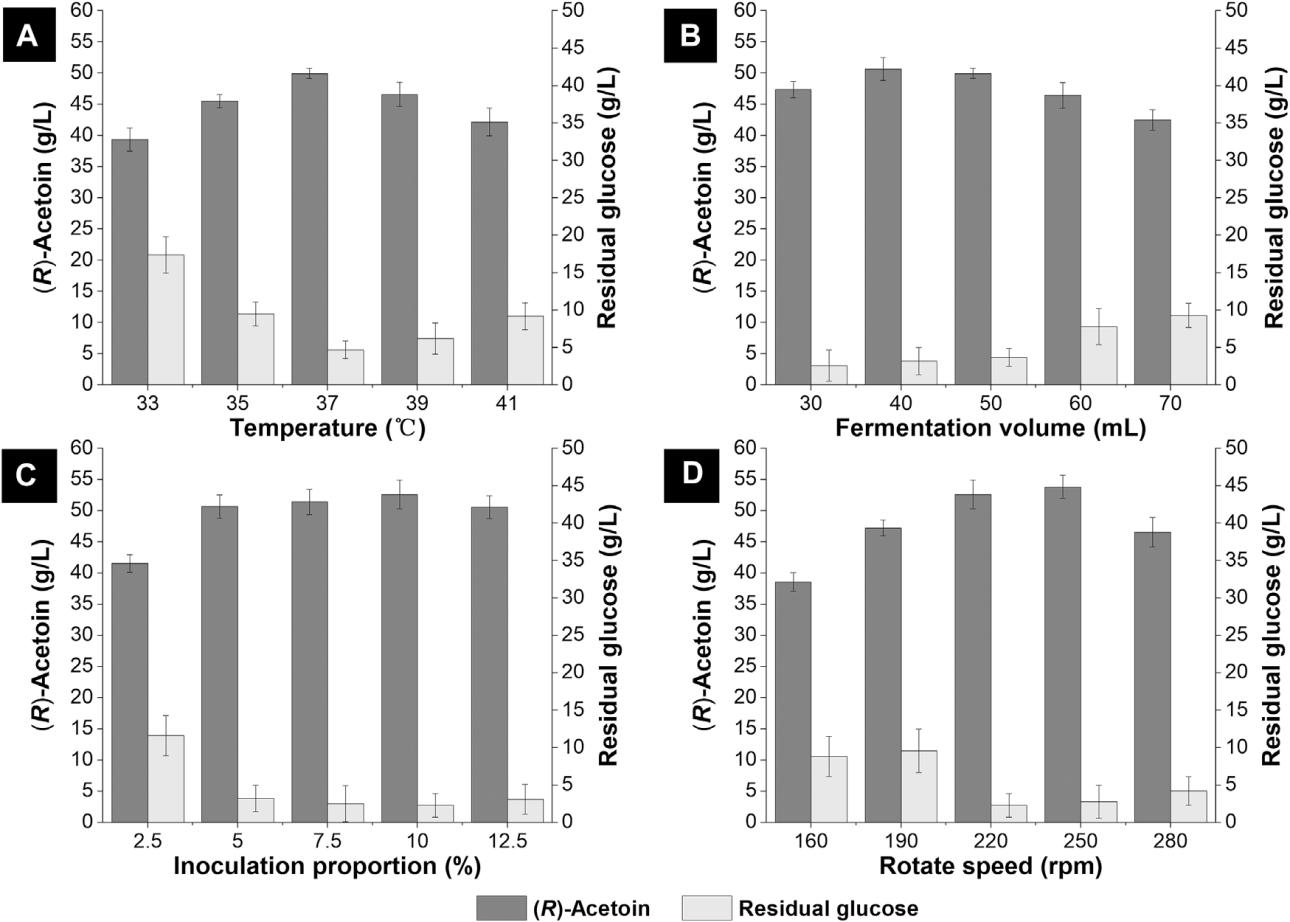
Optimization of the fermentation condition in 250 ml shake flasks. **(A)** temperature (33–41°C) **(B)** fermentation volume (30–70 ml) **(C)** inoculation proportion (2.5–12.5% v/v) **(D)** rotate speed (160–280 rpm).

After optimizing the constituents for the non-food medium, we further evaluated the effects of temperature, fermentation volume, inoculation proportion and rotation speed for (*R*)-AC fermentation in shake flasks. Generally, the optimal growth temperature for *E*. *coli* was about 37°C, but lower or higher temperatures were often employed during the synthesis of certain metabolites. For instance, a titer of (*R*)-AC at 38.3 g/L was achieved by engineered *E. coli* at 35°C in 45 ml fermentation medium (Xu et al., 2015). Even lower temperature (30°C) was employed for AC production with mutant *E. coli* in anaerobic fermentation (Förster et al., 2017). Hence, we investigated the (*R*)-AC production by GXASR11 at 33°C, 35°C, 37°C, 39°C and 41°C, respectively. As seen in Figure 4A, the highest titer of (*R*)-AC was achieved at 37°C and this temperature was found to be the most desirable for cell growth as well (data not shown). The formation of AC by decarboxylation of *α*-acetolactate is an oxidative reaction (Windhorst and Gescher 2019), hence the biosynthesis of AC requires a high level of dissolved oxygen. The generation of 2,3-BD from AC was a reduction reaction (Zhao et al., 2015), therefore a high level of dissolved oxygen in fermentation broth would be beneficial to efficient production of AC while inhibiting the synthesis of 2,3-BD. The fermentation volume and rotation speed influenced the dissolved oxygen in shake flasks. The optimal fermentation volume and rotation speed were found to be 40 ml and 250 rpm, respectively, and resulted in the AC production at 53.83 g/L.

### 3.8 Optimization of Fermentation Condition in 1-L Fermenter

**FIGURE 5:**
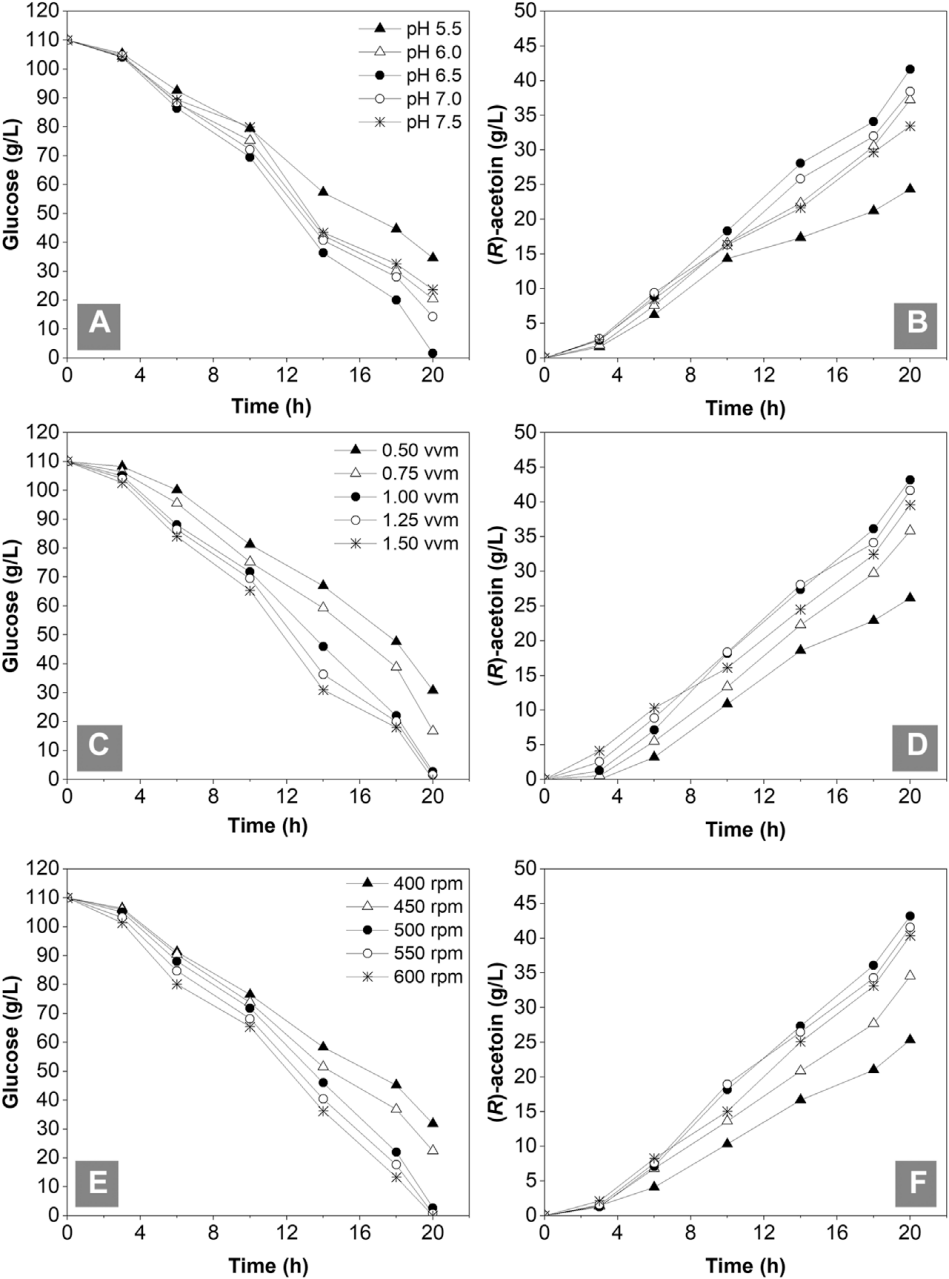
Optimization of initial pH, aeration rates and agitation speed in the 1-L fermenter with an initial broth volume of 0.5 L. **(A)** and **(B)** glucose consumption and (*R*)-AC production under different initial pH from 5.5 to 7.5 **(C)** and **(D)** glucose consumption and (*R*)-AC production under different aeration rates conditions from 0.50–1.50 vvm **(E)** and **(F)** glucose consumption and (*R*)-AC production under different agitation speed conditions from 400 to 600 rpm.

A multiple parallel fermentation system with a 1-L working volume was employed to optimize the initial pH (5.5, 6.0, 6.5, 7.0 and 7.5), aeration rate (0.5, 0.75, 1.0, 1.25 and 1.5 vvm) and agitation speed (400, 450, 500, 500, 550 and 600 rpm) for (*R*)-AC fermentation by GXASR11 at 37°C with a 10% inoculation. Samples were withdrawn at intervals for the determination of residual glucose and (*R*)-AC. The fermentation ceased when the residual glucose declined below 5 g/L. Figures 5A,B showed that the most rapid glucose consumption took place at pH 6.5 with a maximum production of (*R*)-AC at 41.63 g/L. According to the study of Stormer (1968), under slightly acidic condition, the α-acetolactate synthase, which is the key enzyme for synthesis of (*R*)-AC, exhibited higher enzymatic activities and resulted in the accumulation of pyruvic acid. Meanwhile, the activity of lactate dehydrogenase was relatively low, consequently leading to higher amount of pyruvate converted to AC. This observation was consistent with the optimum initial pH reported by previous studies (Dai et al., 2017; Jia et al., 2017b). Moreover, oxygen supply strongly affected the fermentation process of AC (Zhang et al., 2012). According to Figures 5C,D, the glucose consumption, OD_600_ (data not shown) and (*R*)-AC production were all relatively low with an airflow between 0.5 and 0.75 vvm, suggesting that the dissolved oxygen was insufficient for cell growth. The glucose consumption and (*R*)-AC production escalated with increasing ventilation. The highest output of (*R*)-AC (43.18 g/L) and reasonably low by-product content were obtained at 1.0 vvm (data not shown), thus this aeration rate was chosen as the most desirable one. This result is similar to that of a previous study (Xu et al., 2018). The agitation speed of a fermenter not only significantly affected the oxygen level in the fermentation broth, but also the mass transfer efficiency. The (*R*)-AC production and glucose consumption were relatively low when operated at 400 and 450 rpm, suggesting that the efficiency of mass transfer and oxygen supply were insufficient. The glucose consumption at 550 and 600 rpm was close to that at 500 rpm. However, the more oxygen dissolved, the more by-products (e.g., organic acids and alcohols) were produced, which in turn reduced the AC production. Taken together, the agitation speed at 500 rpm was the most desirable for fermentation. As seen in Figures 5B,D and F and Table 4, the production of (*R*)-AC in 1-L fermenter was lower than that in the shake flasks. This was mainly because of a higher level of dissolved oxygen and better mass transfer in shake flasks which led to better cell growth (data not shown) and more glucose consumption.

### 3.9 Fed-Batch Fermentation

**FIGURE 6:**
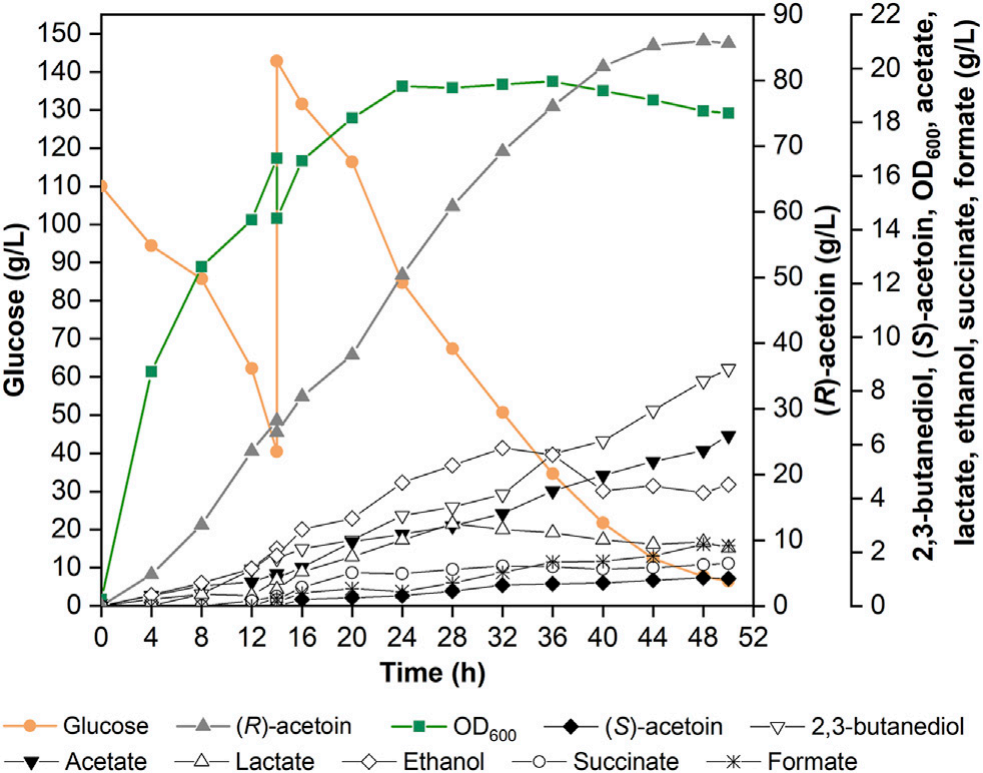
Fed-batch fermentation of (*R*)-AC using 2.5 L non-food feedstocks in a 5-L fermenter by recombinant strain GXASR11 at optimum condition (37°C, inoculation proportion 10%, initial pH 6.5, aeration rate 1.0 vvm, agitation speed 500 rpm).

To boost the production level of (*R*)-AC, the fed-batch fermentation was carried out in a 5-L fermenter with 2.5 L non-food medium. Optimizations of feeding time, concentration and constituents of feeding solution, and feeding rate were carried out for (*R*)-AC production in preliminary experiments (data not shown). The non-food medium contained an initial glucose concentration of 110 g/L and an initial nitrogen concentration of 4.5 g/L. A feeding process was performed when the residual glucose dropped to 40 g/L. The feeding solution containing 800 g/L glucose and 10 g/L nitrogen source was added just once. After the feeding process, the concentration of glucose reached about 140 g/L and the fermentation was not ceased until glucose was completely consumed. As seen in Figure 6, the production of (*R*)-AC peaked at 86.04 g/L after 48 h, then began to decrease while the concentrations of by-products kept increasing. The strategies of fed-batch fermentation or interim feeding were also adopted by other studies with 55.3 g/L and 60.3 g/L productions of AC, respectively (Zhang et al., 2012; Xu et al., 2015).

Several factors could cause the impairment of (*R*)-AC production: 1) in the later stage of fermentation, the catabolism surpassed the anabolism of (*R*)-AC due to the toxicity of accumulated (*R*)-AC to cells (Hosaka et al., 1999; Huang et al., 1999), resulting in a decline of (*R*)-AC concentration; 2) the synthesis of other secondary metabolites, for instance, 2,3-BD, acetate and ethanol, were enhanced; 3) when the carbon and nitrogen sources were insufficient or depleted, (*R*)-AC excreted into the fermentation broth would be catabolized by active cells as the carbon source (Xiao and Xu 2007). Although many previous studies regarding biosynthesis of (*R*)-AC by engineered microorganisms have been reported, a limited number of studies so far were associated with the AC toxicity and microbial resistance against AC (Luo et al., 2014; Li et al., 2015b; Yuan et al., 2019). The mechanism of AC catabolism was only mentioned in several AC-producing *Bacillus* strains (Xiao and Xu 2007), such as *B. subtilis* (Ould Ali et al., 2001), *B*. *licheniformis* (Thanh et al., 2010) and *B*. *thuringiensis* (Peng et al., 2020b). Therefore, in addition to the optimization of the biosynthesis pathway for (*R*)-AC production in *E*. *coli*, the improvement of (*R*)-AC tolerance and the inhibition of its degradation pathways would also be investigated in future research and application.

### 3.10 Condition Optimization of 2,3,5,6-Tetramethylpyrazine Production

The formation of TMP from 2 mol of AC and 2 mol of ammonium is a spontaneous nonenzymatic reaction and is highly influenced by reaction temperature (Xiao et al., 2014; Peng et al., 2020a). It has been observed in many studies that TMP could be spontaneously formed from AC and ammonia salts in water solutions at a slow speed under mild conditions (Besson et al., 1997; Xiao et al., 2018). Noteworthily, the reaction process could be accelerated at elevated temperature (Zhu and Xu 2010a; Xiao et al., 2014). In this study, TMP was synthesized at 180°C to achieve a desirable synthesis efficiency and avoid the accumulation of potential by-products. The precursor AC was enriched in the bio-based stage, for which the optimal fermentation conditions for recombinant *E*. *coli* were determined. In the next stage, the most suitable ammonium salt (DAP) was mixed with fermentation broth for TMP production in a high-pressure microreactor with temperature control. As shown in Table 6, the effects of the molar ratio of AC/DAP, reaction time, temperature and rotation speed of the microreactor on the conversion rate of TMP were investigated respectively. When the reaction was carried out at 90°C and 200 rpm for 2 h with an initial pH of 6.5 at different ratios of precursors (2:5, 2.5:5, 3:5, 3.5:5 and 4:5), the optimum molar ratio of AC/DAP was obtained as 2.5:5 with a maximum conversion rate at 54.20%. The amount of supplemented DAP for higher TMP production was previously investigated (Xiao et al., 2014). It was found that the TMP concentration rose as the AC/ammonium ratio increased from 1/1.0 to 1/2.5, however, further DAP addition could hardly improve the production. The optimal reaction time was identified as 3 h with a conversion rate of 55.15%. Along with the elevation of reaction temperature, the TMP production gradually increased. The most suitable reaction temperature was determined as 180°C and the corresponding TMP production was 45.64 g/L with a conversion rate of 68.64%. The elevated temperature could not only accelerate the formation of TMP in the condensation reaction (Kim et al., 1994), but also cease the further degradation of AC caused by microbial enzymes. Besides, the temperature at 180°C facilitated to minimize the risk of biological pathogenic factors from fermentation (Xiao et al., 2014). Finally, the optimal rotation speed was determined as 400 rpm with an optimal conversion rate of 69.9%.

**TABLE 6:** Optimization of conditions for TMP production (mean ± SD) in microreactor.

Reaction parameters	Production of TMP (g/L)	Conversion rate (%)
Molar concentration ratio		
2:5	33.72 ± 1.43	50.72 ± 2.15
2.5:5	36.04 ± 0.74	54.20 ± 1.11
3:5	33.79 ± 0.78	50.81 ± 1.17
3.5:5	33.46 ± 0.14	50.32 ± 0.22
4:5	29.99 ± 1.01	45.10 ± 1.52
Time (h)		
1	33.19 ± 0.07	49.91 ± 0.10
2	36.04 ± 0.74	54.20 ± 1.11
3	36.67 ± 0.56	55.15 ± 0.84
4	32.58 ± 0.41	48.99 ± 0.62
5	30.77 ± 0.18	46.27 ± 0.27
Temperature (°C)		
90	36.67 ± 0.56	55.15 ± 0.84
120	37.46 ± 1.25	56.33 ± 1.88
150	40.58 ± 2.02	61.03 ± 3.04
180	45.64 ± 1.61	68.64 ± 2.42
210	42.06 ± 0.34	63.25 ± 0.51
Rotation speed (rpm)		
200	45.64 ± 1.61	68.64 ± 2.42
400	46.48 ± 0.38	69.90 ± 0.57
600	44.17 ± 0.77	66.43 ± 1.16
800	42.77 ± 0.48	64.32 ± 0.73
1,200	40.23 ± 0.34	60.50 ± 0.51

SD, standard deviation, n = 3.

**FIGURE 7:**
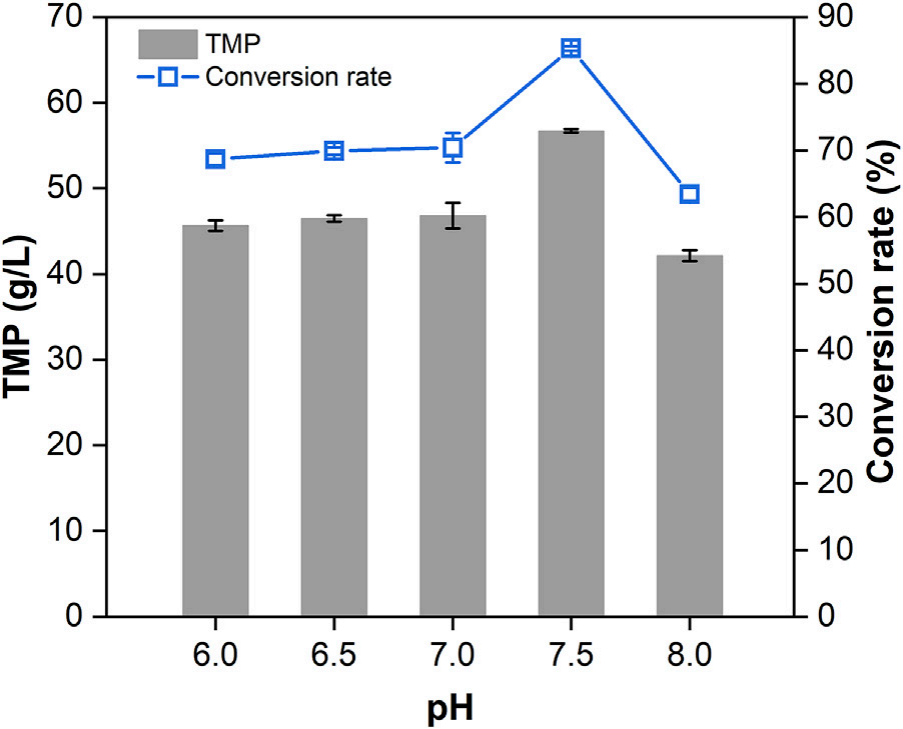
Effect of initial pH (6.0–8.0) on the production of TMP. Error bars represent the standard deviations.

Based on the optimal conditions for TMP conversion (AC/DAP ratio = 2.5:5, reaction time = 3 h, temperature = 180°C and ration speed = 400 rpm), the effect of initial pH on the condensation of TMP was investigated. The pH of fermentation broth was adjusted by phosphoric acid and ammonia solution to 6.0, 6.5, 7.0, 7.5 and 8.0, respectively. The TMP production was determined by GC and the conversion rate was calculated. The results (Figure 7) showed that both the titer and conversion rate of TMP increased with an elevation in pH. The production of TMP peaked at 56.72 g/L at pH 7.5 with a corresponding conversion rate of 85.30%. According to previous reports, the initial pH of fermentation broth towards neutrality or weak alkalinity would be more beneficial for TMP production (Xiao et al., 2006; Zhu and Xu 2010b; Zhu et al., 2010; Zhang et al., 2019). Our observation was consistent with the pH choice at 7.5 from another report (Xu et al., 2018). As the pH increased to 8.0, the titer of TMP began to decline. This was presumably due to the hindrance of surplus NH_4_^+^ and the detailed reasons should be further investigated. Although TMP could be efficiently produced from AC and DAP in a high-pressure microreactor without loss caused by sublimation, the pigments in TMP resulting from the Maillard reaction would complicate the subsequent processing. Thus, effective decolorization methods are required to be exploited for acquiring pure TMP crystal in future work.

## 4 Conclusion

Low cost and green technologies to produce valuable TMP are urgently required to meet the increasing market demand. We have addressed this by construction and optimization of *E. coli* cell factories for high-yield production of (*R*)-AC, and 86.04 g/L (*R*)-AC was obtained using the hydrolysates of tapioca flour and cottonseed cake as cost-effective raw materials by fed-batch fermentation. Subsequently, 56.72 g/L TMP was obtained from DAP and biosynthesized (*R*)-AC in a 20 ml high-pressure microreactor system with a substrate conversion rate of 85.30%. Collectively, this study demonstrated the feasibility that obtaining high-level (*R*)-AC with high optical purity by metabolic engineering and fermentation engineering technology and provided an efficient and sustainable approach for synthesizing high-value TMP via using low-cost biomass feedstocks, making it more economically viable for a scale-up. Further studies will focus on hydrolyzation process optimization, strain engineering for (*R*)-AC resistance and subsequent processing for separation and decolorization of TMP.

## Data Availability

The original contributions presented in the study are included in the article/Supplementary Material, further inquiries can be directed to the corresponding authors.
